# The role of osteomodulin on osteo/odontogenic differentiation in human dental pulp stem cells

**DOI:** 10.1186/s12903-018-0680-6

**Published:** 2019-01-22

**Authors:** Wenzhen Lin, Li Gao, Wenxin Jiang, Chenguang Niu, Keyong Yuan, Xuchen Hu, Rui Ma, Zhengwei Huang

**Affiliations:** 1 Department of Endodontics, Shanghai Ninth People’s Hospital, College of Stomatology, Shanghai Jiao Tong University School of Medicine, Shanghai, China; 2 National Clinical Research Center for Oral Diseases, Shanghai, China; 3 Shanghai Key Laboratory of Stomatology & Shanghai Research Institute of Stomatology, Shanghai, China

**Keywords:** Human dental pulp stem cells, Osteomodulin, Osteo/odontoblastic differentiation

## Abstract

**Background:**

Extracellular matrix secretion and odontoblastic differentiation in human dental pulp stem cells (hDPSCs) are the cellular bases for reparative dentinogenesis. Osteomodulin (OMD) is a member of the small leucine-rich proteoglycan family distributed in the extracellular matrix but little is known about its role in osteo/odontogenic differentiation. The objective of this study was to investigate the role of OMD during osteo/odontoblastic differentiation of hDPSCs.

**Methods:**

hDPSCs were selected using immune-magnetic beads and their capability of multi-differentiation was identified. OMD knockdown was achieved using short hairpin RNA (shRNA) lentivirus and was confirmed by western blot. Gene expression was measured by real-time qPCR and osteo/odontoblastic differentiation of hDPSCs was determined by alizarin red S staining.

**Results:**

Compared with uninduced cells, the transcription of OMD was up-regulated by 35-fold at the late stage of osteo/odontogenic differentiation. shRNA-mediated gene silencing of OMD decreased the expression of odontoblastic genes, such as alkaline phosphatase (ALP), dentin matrix acidic phosphoprotein 1 (DMP1) and dentin sialophosphoprotein (DSPP). Besides, knockdown of OMD attenuated the mineralized nodules formation induced by osteo/odontogenic medium.

**Conclusions:**

These results implied that OMD may play a pivotal role in modulating the osteo/odontoblastic differentiation of hDPSCs.

## Background

The dental pulp contains a unique precursor population of mesenchymal stem cells (MSCs) [1]. MSCs are multipotent, highly proliferative and have the ability to differentiate into odontoblast/odontoblast-like cells in response to the stimuli such as caries or dental trauma [2, 3]. The odontoblasts can secret reactionary/reparative dentine matrix which underlies the formation of dentinal bridge. Cultured dental pulp stem cells can also differentiate into odontoblast-like cells and form calcium nodules under certain circumstances in vitro [4, 5]. Identification of the factors that regulate these processes is of instructive significance.

Dentinogenesis is highly regulated by the expression of the extracellular matrix (ECM) proteins. The dentin contains structural macromolecules and other proteins as extracellular matrix components, including type I collagen, osteonectin, osteopontin and dentin sialoprotein [6, 7]. An important family of molecules with regulatory functions is the small leucine-rich proteoglycans (SLRPs), which are extensively involved in the dentinal biomineralization [8]. In particular, it has been confirmed that biglycan and decorin are identified in the matrices of dentin and implicated in dentinogenesis [9].

Osteomodulin (OMD), a heterologous protein of Osteoadherin, belongs to SLRPs and was originally isolated as a keratan sulfate proteoglycan from bovine long bone [10]. SLRPs normally distribute in extracellular matrices, but OMD is the only member specifically restricted to mineralized tissues [11, 12]. Not only does OMD have a high affinity for hydroxyapatite crystals via its large and acidic C-terminal domain [10, 13], but also it can directly regulate diameter and alter shape of type I collagen fibrils [14]. However, the functions of OMD on osteo/odontoblastic differentiation and mineralization have yet to be fully determined, nevertheless it has been shown that OMD expression starts in the polarized odontoblasts and increases in the odontoblast cell layer and alveolar bone during early crown formation [15, 16]. Therefore, it was hypothesized that OMD may be positively correlated with the osteo/odontogenic differentiation and accordingly, the purpose of the present study was to investigate the influence of OMD deficiency on the biomineralization of hDPSCs.

## Methods

### Isolation and culture of hDPSCs

Healthy human third molars extracted for orthodontic treatment purpose were obtained from 17- to 20-year-old individuals at the oral surgery clinic of the Ninth People’s Hospital affiliated to Shanghai Jiao Tong University School of Medicine. The primary cultured human dental pulp cells were isolated from ten molars using explant method. The cells were pooled together to select hDPSCs by STRO-1-labelled magnetic beads as described previously [17, 18]. Cells were cultured in growth medium (GM): high-glucose Dulbecco’s modified Eagle’s medium (DMEM; Gibco-BRL, Grand Island, NY, USA) supplemented with 10% fetal bovine serum (Gibco-BRL, Life Technologies, Paisley, UK), 100 U/mL penicillin, and 100 mg/mL streptomycin. The medium was renewed every 2 or 3 days. Cell cultures between the second and fifth passages were used.

### Flow cytometric analysis

The cell surface markers present on hDPSCs were detected by flow cytometric analysis [19, 20]. Briefly, hDPSCs were incubated with fluorescence-conjugated antibodies for CD73-phycoerythrin (PE), CD105-PE, CD166-PE, CD34-PE, CD90-fluorescein iso thioocyanide (FITC) and CD45-FITC (BD Biosciences, San Jose, CA, USA, and Biolegend, San Diego, CA, USA). Cell suspensions in phosphate buffered saline (PBS) without antibodies served as controls. The cells were then washed three times with PBS to remove unbound antibodies and finally resuspended with 300 μL PBS. Cells were sorted using a flow cytometer (FACSCalibur; BD Biosciences, Mountain View, CA, USA) and analysed with FlowJo software (Tree Star, San Carlos, CA, USA).

### Alizarin red S staining and oil red O staining assay

For osteo/odontogenic differentiation, hDPSCs were subcultured in human mesenchymal stem cell osteogenic differentiation medium (OM) (Cyagen Biosciences, Santa Clara, CA, USA) which contained dexamethasone, L-ascorbic acid and beta-glycerophosphate for up to 21 days. Cells cultured in GM were kept as a control group. After 3 weeks of differentiation, cells were fixed in 4% paraformaldehyde for 30 min. After being washed with PBS for three times, calcium deposition was visualized by staining with alizarin red S solution for 3–5 min. Excess stain was removed by washing with distilled water. To study adipogenesis, hDPSCs were cultured in GM until they reached 100% confluence, following which the medium was changed to adipogenic differentiation medium (AM) (Cyagen Biosciences) which contained Insulin, IBMX, Rosiglitazone and Dexamethasone according to the manufacturer’s instructions. After three to five cycles of induction/maintenance, cells were fixed in 4% paraformaldehyde for 30 min and stained with fresh oil red O solution.

### Lentivirus production and transduction

Sequences for constructing shRNA targeting human OMD were obtained from the RNAi Consortium (Broad institute) [21]. shRNAs against the OMD gene and a non-target gene were generated with PLKO.1 vector (sequences were shown in Table 1). 9 μg of the ViralPower™ Packaging Mix (Invitrogen, Carlsbad, CA, USA) and 3 μg of the constructed PLKO.1-shOMD or PLKO.1-Ctrl vector were used to co-transfect 6 × 10^6^ 293FT cells in the presence of 36 μL Lipofectamine™ 2000 (Invitrogen). Lentivirus was harvested from the culture supernatant at 48 h and 72 h after transfection and filtered through a 0.45 μm filter. For infection, hDPSCs cultured to 30–40% confluence were exposed to recombinant lentivirus in the presence of 10 μg/mL polybrene for 24 h. After incubated in GM for another 24 h, cells were treated with 1 μg/mL puromycin for 48 h to generate stable cell lines.

**Table 1:** Primer sequences

Gene	Primer sequences
For lentiviral constructs	
shOMD_F	5’-CCGGGATCACGATGATCCTGACAATCTCG -AGATTGTCAGGATCATCGTGATCTTTTTG-3’
shOMD_R	5’-AATTCAAAAAGATCACGATGATCCTGACA -ATCTCGAGATTGTCAGGATCATCGTGATC-3’
non-target shRNA_F	5’-CCGGCAACAAGATGAAGAGCACCAACTC -GAGTTGGTGCTCTTCATCTTGTTGTTTTTG-3’
non-target shRNA_R	5’-AATTCAAAAACAACAAGATGAAGAGCAC CAACTCGAGTTGGTGCTCTTCATCTTGTTG-3’
For real-time qPCR and RT-PCR	
OMD_F	5′- AGGCTGTGTCAGTGAATGCT-3’
OMD_R	5′- GTTGCTGAATGTGCATCGGA-3’
DSPP_F	5’-GCCATTCCAGTTCCTCAAAGC-3’
DSPP_R	5’-CATGCACCAGGACACCACTT-3’
DMP 1_F	5’-ATGCCTATCACAACAAACC-3’
DMP 1_R	5’-CTCCTTTATGTGACAACTGC-3’
ALP_F	5’-GGACCATTCCCACGTCTTCAC-3’
ALP_R	5’-CCTTGTAGCCAGGCCCATTG-3’
β-actin_F	5’-TGGCACCCAGCACAATGAA-3’
β-actin_R	5’-CTAAGTCATAGTCCGCCTAGAAGCA-3’

### Western blot analysis

Western blot was performed as described before [22]. Briefly, cells were washed with PBS and harvested with EBC lysis buffer (50 mM Tris HCl, pH 8.0, 120 mM NaCl, 0.5% Nonidet P-40) supplemented with protease inhibitors (Selleck Chemicals, Houston, TX, USA). Protein fractions were collected by centrifugation at 10,000 g at 4 °C for 10 min. The supernatants which contained 50 μg of protein samples were subjected to 10% sodium dodecyl sulfate polyacrylamide gel electrophoresis, following protein quantitation using BCA protein assay. Protein was transferred onto the polyvinylidene difluoride membrane. After blocking in 1 × TBS-0.05%Tween 20 (TBST) containing 5% dry milk powder, membranes were incubated with OMD antibodies (1: 1000; Abgent, San Diego, CA, USA) and α-tubulin antibodies (1: 1000; Santa Cruz Biotechnology, Santa Cruz, CA, USA) overnight at 4 °C. After the membranes were incubated with HRP-conjugated secondary antibody (DAKO, Glostrup, Denmark) for 1 h and extensive washing with 1 × TBST, specific bands were reacted with an ECL chemiluminescence detection system (Thermo Fisher Scientific, Waltham, MA, USA) and visualized with X-ray films (Carestream, Xiamen, Fujian, China).

### RNA isolation and determination

Total RNA was extracted at designated time points using TRIzol reagent (Invitrogen) according to the manufacturer’s instructions. Then, 500 ng extracted RNA was reverse transcribed using Ommiscript Reverse Transcription kit (QIAGEN, Valencia, CA, USA) and the resulting cDNA was diluted to 5 ng/μL. 1 μL of this diluted product was used as a template for real-time quantitative PCR (real-time qPCR) and 0.5 μL for reverse transcription PCR (RT-PCR). Real-time qPCR was performed using the DNA Engine OPTICON™ 2 system (MJ Research, Waltham, MA, USA) with SYBR Green I fluorogenic dye (Molecular Probes, Eugene, OR, USA) under the following conditions: 40 cycles each involving 5 s of denaturation at 95°C and 30 s of amplication at 60°C. RT-PCR was performed using Go Taq1 Flexi DNA polymerase (Promega, Madison, WI, USA) under the following conditions: 25 cycles each involving 30 s of denaturation at 94°C, 30 s of annealing at 55°C and 20 s of extension at 72°C. The primers were listed in Table 1. The mRNA levels of target genes were analysed according to the comparative Cq method [23] and normalized to β-actin.

### Statistical analysis

Data were expressed as means ± standard deviation (SD) from at least three independent experiments. The statistical significance of difference was assessed using Student’s two-tailed *t-*test. *P* < 0.05 indicated a significant difference between groups.

## Results

### Characterization of the hDPSCs

**Fig. 1:**
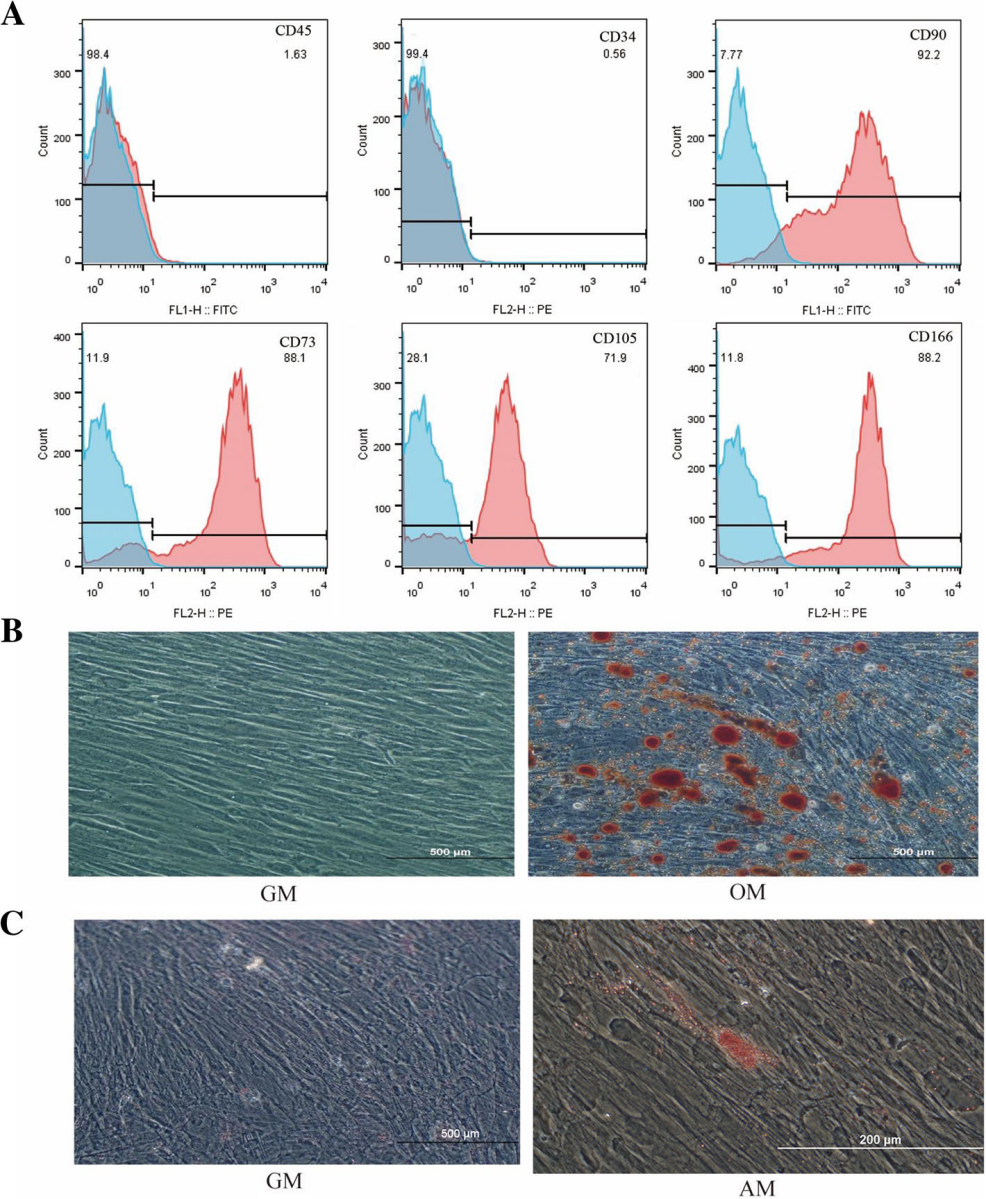
The characteristics of hDPSCs. **a** Surface marker distribution on human dental pulp stem cells. **b** The results of alizarin red S staining. **c** The results of oil red O staining. GM: growth medium; OM: osteo/odontogenic induction medium; AM: adipogenic induction medium

The hDPSCs possess many in vitro phenotypic characteristics of bone marrow-derived MSCs [24, 25]. Flow cytometric analysis was one of the methods based on cell surface molecules. hDPSCs showed the characteristic pattern of MSC-associated surface markers, including CD73, CD90, CD105 and CD166 and were negative for hematopoietic stem cell surface markers CD34 and CD45. Isolated cells that highly expressed CD73, CD90, CD105 and CD166 were used for subsequent experiments (Fig. 1a). The hDPSCs retained multilineage differentiation capacity. Calcium deposition was confirmed by alizarin red S staining (Fig. 1b) and lipid formation was revealed by oil red O staining (Fig. 1c), which indicated hDPSCs’ differentiation into cells like osteoblasts and adipocytes.

### Up-regulation of OMD during osteo/odontogenic differentiation

**Fig. 2:**
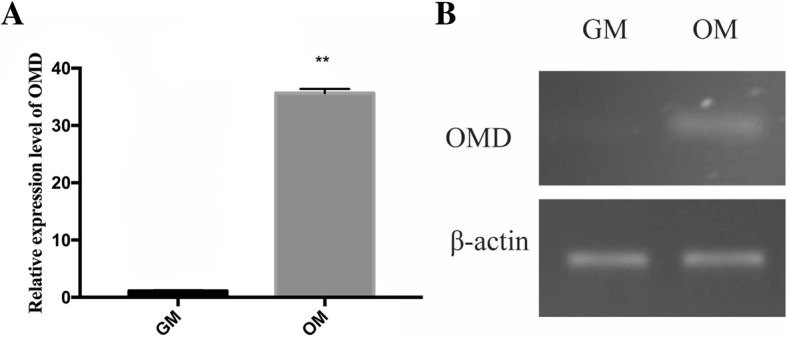
The transcription level of the OMD gene in DPSCs before and after osteo/odontogenic induction by real-time qPCR (**a**) and RT-PCR (**b**). **Significant difference (*P* < 0.01) versus Ctrl

To investigate the expression pattern of OMD mRNA during osteo/odontogenic differentiation, hDPSCs were cultured in OM for 3 weeks, after which samples were analysed by real-time qPCR and RT-PCR. Compared with the control group in GM, hDPSCs which were incubated in induction medium showed a significant 35-fold up-regulation in OMD gene expression (Fig. 2).

### Knockdown of OMD in hDPSCs

**Fig. 3:**
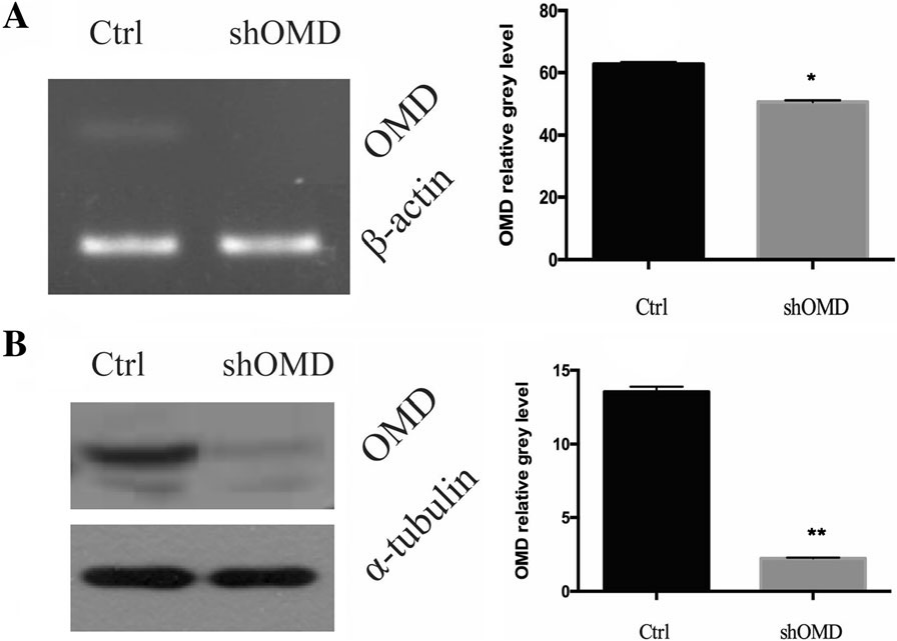
The identification of shOMD-hDPSCs cultured in GM. **a** The transcription of OMD gene in Ctrl-hDPSCs and shOMD-hDPSCs by RT-PCR. **b** The expression of OMD protein in Ctrl-hDPSCs and shOMD-hDPSCs by western blot. Ctrl: the control group; shOMD: the OMD knockdown group. *Significant difference (*P* < 0.05) versus Ctrl. **Significant difference (*P* < 0.01) versus Ctrl

Puromycin treatment for 2 days almost completely eliminated uninfected hDPSCs without affecting the growth rate and morphology of successfully infected hDPSCs. hDPSCs which were infected with lentiviral constructs harbouring OMD shRNA and non-target shRNA could survive puromycin treatment and were indicated as shOMD-hDPSCs and Ctrl-hDPSCs, respectively. The efficiency of the shRNA-mediated knockdown was confirmed by real-time qPCR and Western blot without induction. The results showed that OMD mRNA in shOMD-hDPSCs was reduced with a concomitant decrease in OMD protein levels in GM (Fig. 3a and b).

### Inhibition of OMD impairs osteo/odontoblastic differentiation of hDPSCs

**Fig. 4:**
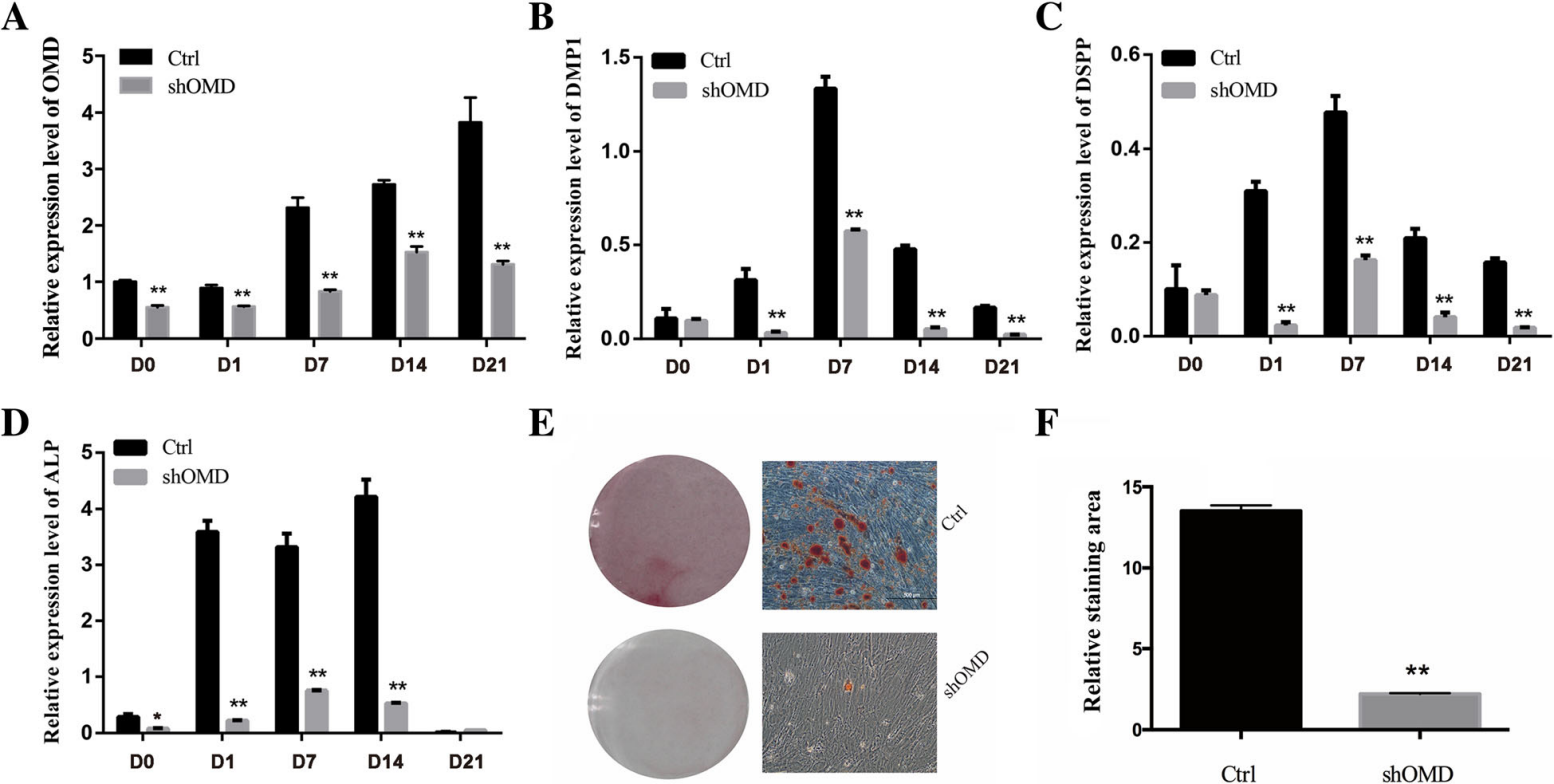
The mRNA levels of **a** OMD, **b** DMP1, **c** DSPP and **d** ALP in Ctrl-hDPSCs and shOMD-hDPSCs during osteo/odontogenic induction by real-time qPCR. **e** Alizarin red S staining in Ctrl-hDPSCs and shOMD-hDPSCs after induction. **f** The relative quantification of the mineralization nodules in (**e**). **Significant difference (*P* < 0.01) versus Ctrl

The stable gene knockdown was achieved throughout the differentiation period (Fig. 4a). The transcriptional level of OMD in Ctrl-hDPSCs was progressively increased with induction. The results of real-time qPCR showed that the expression of the odontoblast markers DMP1 and DSPP in Ctrl-hDPSCs were substantially up-regulated during 7 days of induction and ALP mRNA in Ctrl-hDPSCs reached its peak on day 14. As is shown in Fig. 4, the mRNA levels of DMP1, DSPP and ALP in shOMD-hDPSCs were significantly lower than those in the control group at each given time point. Specifically speaking, DMP1, DSPP and ALP mRNA levels in Ctrl-hDPSCs were at least twice as high as those in shOMD-hDPSCs at early induction period. After osteo/odontogenic induction for 14 days, DMP1, DSPP and ALP mRNA levels in Ctrl-hDPSCs were at least three times higher than those in shOMD-hDPSCs. In addition, shOMD-hDPSCs could not form calcified nodules or differentiate into osteo/odontoblast-like cells after 21 days of culture in osteo/odontogenic medium compared with the control groups (Fig. 4e and f).

## Discussion

hDPSCs play an essential role in dentinogenesis and dental repair [26]. The capacity of self-renewal and multilineage differentiation into cell types such as odontoblasts/osteoblasts, adipocytes, and neuron-like cells attracts researchers a lot. hDPSCs have been considered as an alternative therapeutic cell source for dental tissue and whole-tooth regeneration [27]. Therefore, the identification of genes regulating the hDPSCs into osteo/odontogenic fate will help clarify the mechanisms of regenerative strategies.

Heretofore multiple signaling molecules and transcription factors, including bone morphogenetic proteins, fibroblast growth factors, Wnt proteins, Hedgehog families and Cbfa1/Runx2 protein, have been shown to be implicated in mediating differentiation and organization of osteogenic tissues when responding to inductive signals [28]. However, there still existed mechanisms that have not been disclosed. To the best of our knowledge, OMD was one of the genes which had seldomly been investigated with regard to its expression patterns and functions in hDPSCs’ cytodifferentiation.

In this study, the hDPSC subpopulation expressing STRO-1 surface marker was used. In accordance with previous reports [29, 30], the isolated hDPSCs expressed MSC-specific cell surface antigens such as CD73, CD90, CD105 and CD166, and were negative for the hematopoietic surface markers CD34 and CD45. In our previous study, it was found that the expression of STRO-1 declined gradually with the continuing passage of cells (data not shown). Thus cell cultures between the second and fifth passages were used. In this study, mineral nodules (considered as a late marker of osteo/odontogenic differentiation [31]) were found to increase with the induction period of hDPSCs. ALP, DMP1 and DSPP (also referred as osteo/odontogenic differentiation markers [32, 33]) were up-regulated in the cells of the control group during the early induction period.

DSPP is a pre-proprotein secreted by odontoblasts and its cleaved products named dentin sialoprotein and dentin phosphoprotein are found in significant quantity in the extracellular matrix of dentin [34]. DMP1 has been reported to be expressed during the initial stages of mineralized matrix formation in bone and dentin [35]. The expression levels of DSPP and DMP1 suggest the dentinogenesis ability of dental pulp cells (DPCs) [36]. In the studies of Lin et al. and Qi et al., the transcription levels of DSPP and DMP1 increased continuously during odontoblastic differentiation of DPCs and achieved the highest level after 14 days of induction [37, 38], while in this study they reached the highest point at day 7. It is supposed that the difference in induction medium and cellular condition of hDPSCs may explain the discrepancies. Meanwhile, the expression of OMD was up-regulated in mRNA level after 21 days of induction. So it was proposed a hypothesis that OMD may regulate the osteo/odontoblastic differentiation of hDPSCs.

To test our hypothesis, OMD knockdown hDPSCs were established by infecting them with a lentiviral construct harboring shRNA targeting OMD. It is worth noting that the OMD transcriptional level in Ctrl-hDPSCs after induction for 21 days was three times higher than that in Ctrl-hDPSCs cultured on day 0 (Fig. 4a), while the OMD mRNA of the induced and uninfected hDPSCs was around 35 times higher than that in uninduced and uninfected hDPSCs (Fig. 2a). Reasons bringing about this phenomenon may be the lentivirus process and its potential influence on gene expression. Nonetheless, it could be noticed that OMD knockdown dramatically suppressed the differentiation of hDPSCs into osteo/odontoblasts according to the low expression of ALP, DMP1 and DSPP and reduction of calcified nodules formation. Studies have explored the potential mechanisms of OMD and it has been reported that the expression of OMD is regulated by the cytokines TGFβ1 and BMP2: TGFβ1 down-regulates OMD, while BMP-2 up-regulates OMD [11]. Therefore, it is proposed that OMD may plays a certain role in TGFβ and BMP signaling during osteo/odontogenic differentiation. However, the exact function of OMD during mineralization remains to be fully elucidated.

## Conclusions

This study demonstrates that OMD knockdown can inhibit the osteo/odontoblastic differentiation of hDPSCs by suppressing mineralization and the expression of osteo/odontoblast-related genes. OMD may promote the osteo/odontogenic differentiation of hDPSCs. Further investigation is required to elucidate the mechanisms by how OMD regulates the biological characteristics of hDPSCs.

## Abbreviations

ALP: alkaline phosphatase
AM: adipogenic differentiation medium
DMP1: dentin matrix acidic phosphoprotein 1
DSPP: dentin sialophosphoprotein
FITC: fluorescein iso thioocyanide
GM: growth medium
hDPSCs: human dental pulp stem cells
mRNA: messenger ribonucleic acid
OM: osteo/odontogenic differentiation medium
OMD: Osteomodulin
PBS: phosphate buffered saline
PE: phycoerythrin
qPCR: quantitative PCR
RT-PCR: reverse transcription PCR
shRNA: short hairpin RNA
SLRP: small leucine-rich proteoglycan
